# *ADRB2* expression predicts the clinical outcomes and is associated with immune cells infiltration in lung adenocarcinoma

**DOI:** 10.1038/s41598-022-19991-y

**Published:** 2022-09-26

**Authors:** Lingyun Ji, Fei Xu, Jingtao Zhang, Ting Song, Weida Chen, Xi Yin, Qingqing Wang, Xiubao Chen, Xin Li, Minghao Guo, Zetao Chen

**Affiliations:** 1 First Clinical Medical College, Shandong University of Traditional Chinese Medicine, Jinan, China; 2 Department of Geriatric Medicine, Affiliated Hospital of Shandong University of Traditional Chinese Medicine, Jinan, China; 3 College of Traditional Chinese Medicine, Shandong University of Traditional Chinese Medicine, Jinan, China; 4 Department of Neurology, Affiliated Hospital of Shandong University of Traditional Chinese Medicine, Jinan, China; 5 Department of Record Room, Affiliated Hospital of Shandong University of Traditional Chinese Medicine, China Jinan,; 6 Subject of Integrated Chinese and Western Medicine , Shandong University of Traditional Chinese Medicine, Jinan, China

**Keywords:** Cancer, Computational biology and bioinformatics, Immunology, Biomarkers, Oncology

## Abstract

The gene encoding beta2-adrenergic receptor (β2-AR), adrenoceptor beta 2 (*ADRB2*), has been reported to closely associated with various cancers. However, its role in lung adenocarcinoma (LUAD) remains controversial. This research shed light on the prognostic value of *ADRB2* in LUAD and further explored its association with immune cell infiltration. *ADRB2* was significantly decreased in LUAD. *ADRB2* expression in LUAD was significantly correlated with gender, smoking status, T classification, and pathologic stage. Patients in the low *ADRB2* expression group presented with significantly poorer overall survival (OS) and disease-specific survival (DSS). Kyoto Encyclopedia of Genes and Genomes (KEGG) and Gene Set Enrichment Analysis (GSEA) results showed that *ADRB2* participates in immune response. The expression of *ADRB2* was positively correlated with the infiltration level of most immune cells. Notably, *ADRB2* is involved in LUAD progression partly by regulating the immune microenvironment, which may potentially serve as a significant prognostic biomarker as well as a potential drug target.

## Introduction

Lung cancer is the primary cause of malignant tumor mortality globally^1^. LUAD, one of the highest mortality rates and most aggressive forms of cancer, with a low 5-year survival rate < 5%^2^. Late diagnosis may lead to difficulties in the treatment and prediction of prognosis. Thus, an in-depth study of the molecular mechanisms underlying LUAD progression is urgently needed. At present, there remains an unmet clinical need for tumor biomarkers, and the search for these could lead to more effective treatments and longer survival.

*ADRB2* encodes beta-2-adrenergic receptor (β2-AR) which is a member of the G protein-coupled receptor superfamily (GPCRs). GPCRs consist of a large family of integral membrane proteins with seven transmembrane helices. Adrenergic receptors (ARs), a member of GPCRs, are classically divided into two main groups: α-and β-adrenoceptors (β-AR, which is divided into β1, β2, and β3 subtypes)^3^. β-AR could facilitate cell proliferation, migration, invasion, inflammation, angiogenesis, apoptosis, cell immune response, and epithelial-mesenchymal transition by regulating multiple cancer-related cellular processes. Dysregulated expression of *ADRB2* was observed in various cancers, including breast cancer^4^, hepatocellular carcinoma^5^, prostate cancer^6^, and ovarian carcinoma^7^. Moreover, abundant *ADRB2* expression was found to be closely linked with poor clinicopathological characteristics, tumor recurrence, metastasis, and poor prognosis. Although *ADRB2* is a carcinogenic biomarker; however, the clinical significance of its expression in patients with LUAD has not been thoroughly elucidated yet.

It is well known that the tumorigenesis, progression, OS, prognosis, and relapse of tumors are strongly linked to the expression of tumor genes. The gene encoding β2-AR, *ADRB2*, maps to human chromosome 5q31–q32 and is composed of a single exon of 2015 nucleotides^8^. The effect of *ADRB2* on lung cancer remains controversial. Mei et al.^9^ identified *ADRB2* polymorphisms that were correlated with increased lung cancer risk. Nevertheless, Zheng et al.^1^ found that *ADRB2* was underexpressed in LUAD tissues and low *ADRB2* expression is associated with poor clinical outcomes. The other research by Wang et al. reached the same conclusion^10^.

Based on The Cancer Genome Atlas (TCGA) dataset, LUAD dataset was acquired for bioinformatics analysis to verify that *ADRB2* expression was significantly down-regulated in LUAD. Next, the relationship between *ADRB2* gene expression and clinical traits was further investigated. The expression of *ADRB2* was highly correlated with immune infiltration, which further confirmed that *ADRB2* could be used as a prognostic biomarker of LUAD.

## Results

### Patient characteristics

The clinical data of 535 LUAD patients included gender, age, smoking status, T stage, N stage, M stage, pathological stage, OS event, and DSS event (Table 1). Chi-square test revealed that *ADRB2* was significantly correlated with T stage (P < 0.001), gender (P = 0.005), smoking (P = 0.004), OS events (P < 0.001) and DSS events (P = 0.005). There was no significant correlation between *ADRB2* expression and other clinicopathological features.

**Table 1:** Demographic and clinicopathological parameters of high and low *ADRB2* expression group patients with lung adenocarcinoma in TCGA-LUAD.

Characteristic	Low expression of *ADRB2*	High expression of *ADRB2*	P
n	267	268	
**Gender, n (%)**			0.005
Female	126 (23.6%)	160 (29.9%)	
Male	141 (26.4%)	108 (20.2%)	
**Age, n (%)**			0.723
< = 65	131 (25.4%)	124 (24%)	
> 65	129 (25%)	132 (25.6%)	
**Smoker, n (%)**			0.004
No	25 (4.8%)	50 (9.6%)	
Yes	233 (44.7%)	213 (40.9%)	
**T stage, n (%)**			< 0.001
T1	68 (12.8%)	107 (20.1%)	
T2	151 (28.4%)	138 (25.9%)	
T3	33 (6.2%)	16 (3%)	
T4	13 (2.4%)	6 (1.1%)	
**N stage, n (%)**			0.299
N0	167 (32.2%)	181 (34.9%)	
N1	52 (10%)	43 (8.3%)	
N2	43 (8.3%)	31 (6%)	
N3	1 (0.2%)	1 (0.2%)	
**M stage, n (%)**			1.000
M0	187 (48.4%)	174 (45.1%)	
M1	13 (3.4%)	12 (3.1%)	
**Pathologic stage, n (%)**			0.052
Stage I	132 (25%)	162 (30.7%)	
Stage II	66 (12.5%)	57 (10.8%)	
Stage III	51 (9.7%)	33 (6.3%)	
Stage IV	14 (2.7%)	12 (2.3%)	
**OS event, n (%)**			< 0.001
Alive	152 (28.4%)	191 (35.7%)	
Dead	115 (21.5%)	77 (14.4%)	
**DSS event, n (%)**			0.005
Alive	173 (34.7%)	206 (41.3%)	
Dead	73 (14.6%)	47 (9.4%)	

### *ADRB2* expression level in LUAD

**Figure 1:**
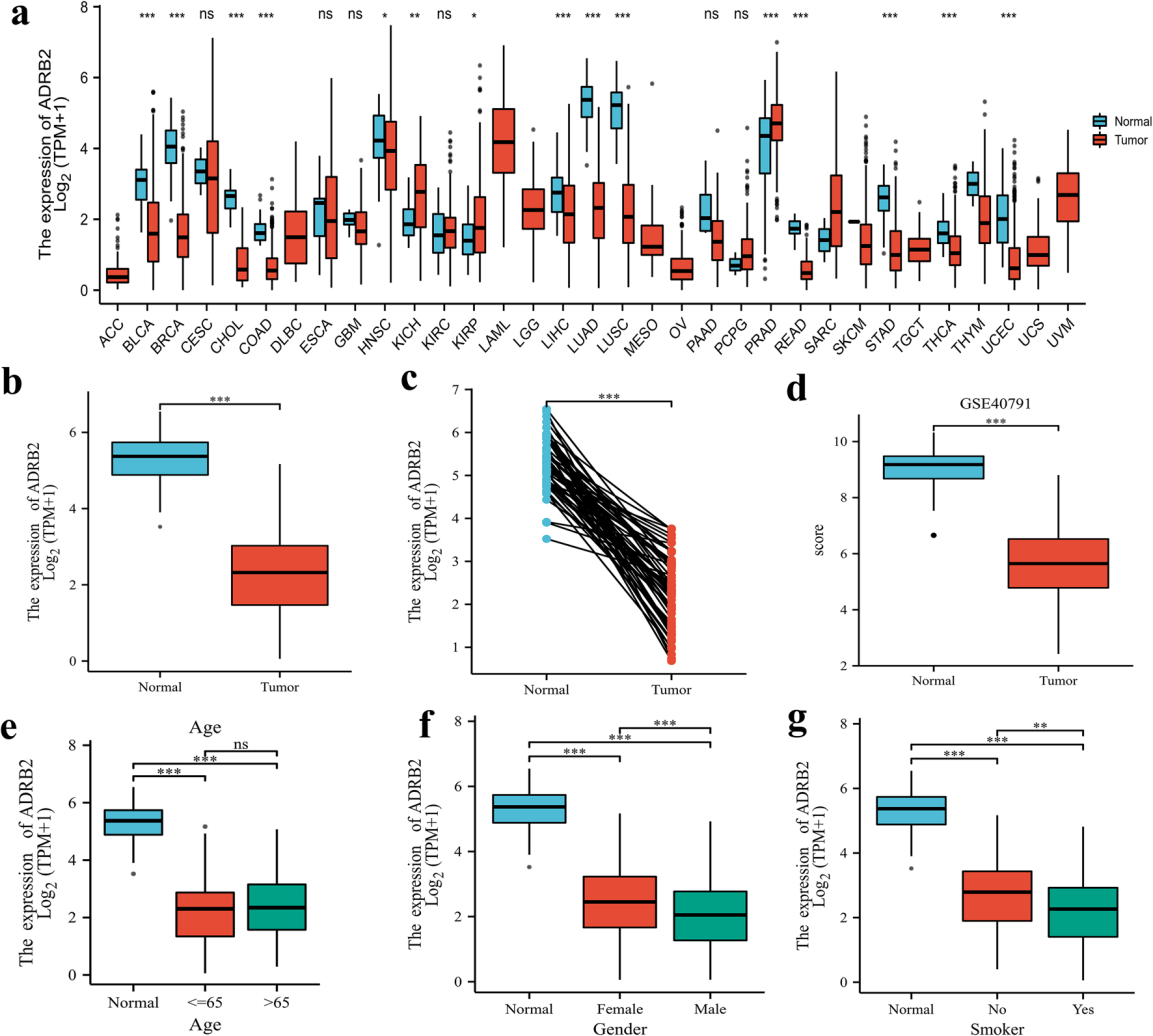
Relative expression level of *ADRB2* in LUAD from TCGA database and GEO database. (**a**) *ADRB2* mRNA expression levels in pan-cancers from TCGA database. (**b**) Boxplot of *ADRB2* expression between the LUAD and normal tissues from TCGA database (Normal = 59 and Tumor = 535). (**c**) Pairwise boxplot of *ADRB2* expression between the LUAD and normal tissues from TCGA database (Normal = 57 and Tumor = 57). The differential expression of *ADRB2* in GSE40791 from GEO database (Normal = 100 and Tumor = 96) (**d**).The expression of *ADRB2* is grouped by age (**e**), gender (**f**), and smoking status (**g**). ***P < 0.001, *ns* not significance.

Based on the TCGA database, *ADRB2* mRNA expression level was analyzed in 594 tissues. As showcased in Fig. 1a, it evaluated the *ADRB2* mRNA expression levels in TCGA pan-cancer. The results indicated that the expression level of *ADRB2* in the LUAD samples was much higher than that in the normal tissue samples (P < 0.001). Box plots showed *ADRB2* mRNA expression levels in 59 adjacent non-tumor tissues and 535 LUAD tissues. As shown in Fig. 1b, *ADRB2* was down-expression in LUAD tissues compared with those in normal tissues (P < 0.001, Fig. 1b). Figure 1c shows a pairwise boxplot of the same outcomes (P < 0,001). Figure 1d shows 100 normal samples and 94 LUAD samples from the GEO database, which shows that *ADRB2* is lowly expressed in LUAD patients (P < 0,001). *ADRB2* expression in LUAD patients does not show correlation to age (Fig. 1e). Moreover, *ADRB2* was significantly lower in males (P < 0.001, Fig. 1f) and in patients with a smoking history (P < 0.001, Fig. 1g).

### Association between *ADRB2* and TNM stages in LUAD patients

**Figure 2:**
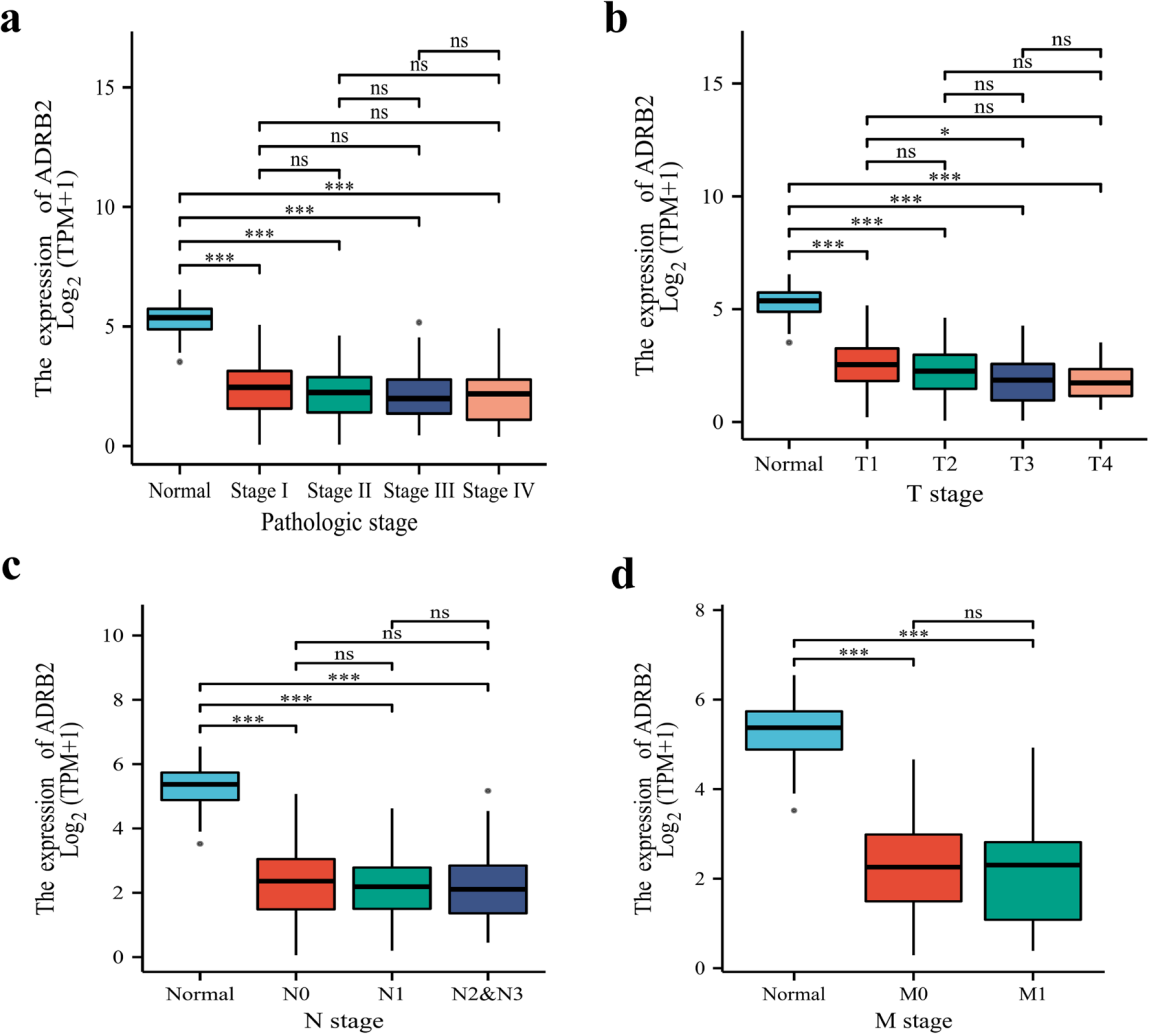
The expression of *ADRB2* is grouped by pathological stage (**a**), T stage (**b**), T stage (**c**), and M stage (**d**). *P < 0.05, **P < 0.01, ***P < 0.001, *ns* not significance.

To better understand the impact of *ADRB2* on LUAD patient prognosis, Kruskal–Wallis analysis and Spearman correlation analysis were performed to determine the relationship between *ADRB2* expression and clinicopathological characteristics (pathologic and TNM stages). The *ADRB2* expression was significantly decreased in LUAD patients, and was significantly correlated with pathological stage, T stage and N stage. (Fig. 2a–d, Supplementary Table S1).

### Relationship between *ADRB2* and clinical characteristics

**Figure 3:**
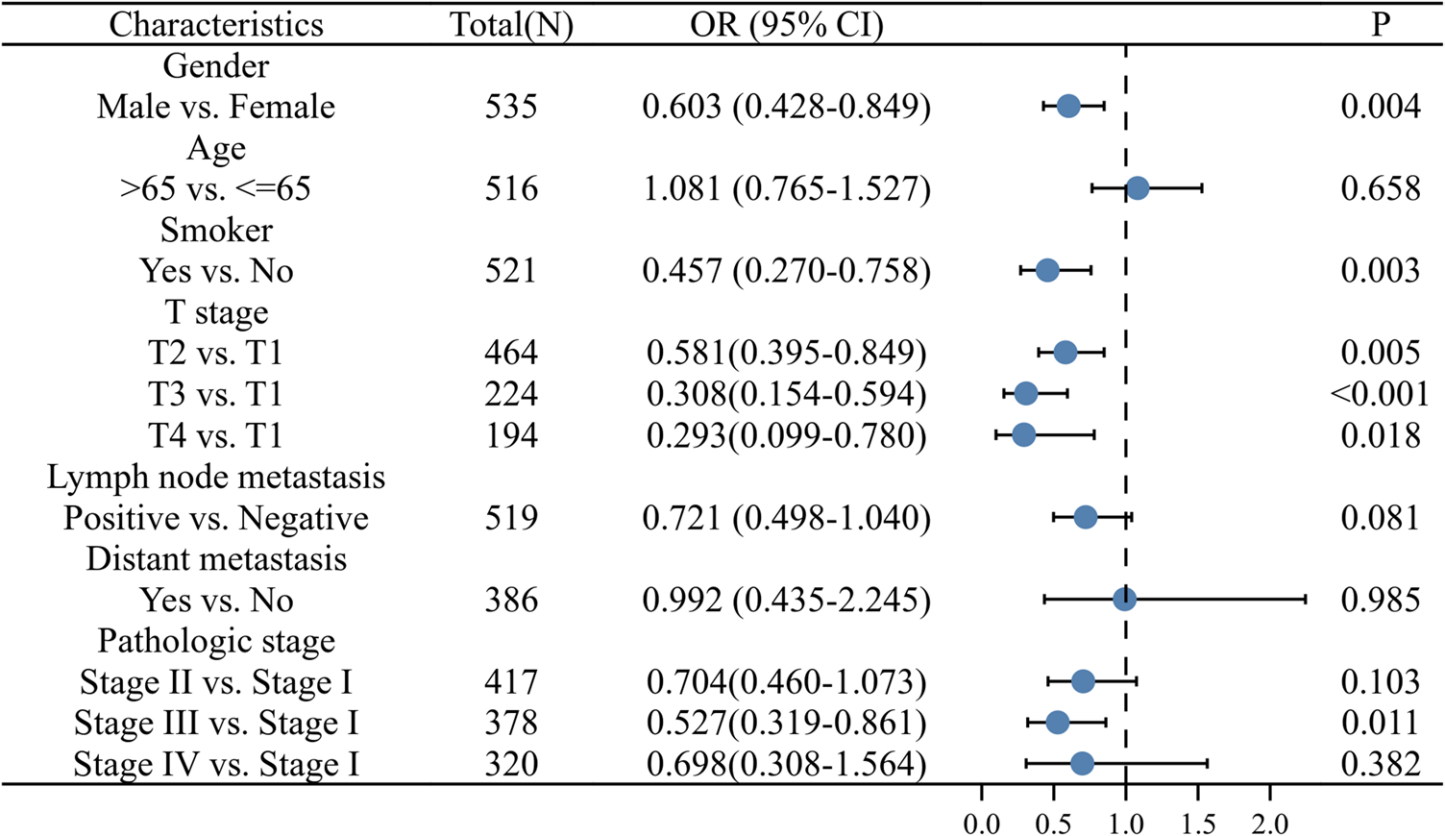
Relationship between *ADRB2* and clinical characteristics.

To further investigate the mechanism of *ADRB2* in LUAD, the associations between *ADRB2* expression and clinical characteristics were investigated. Based on the clinical data of 535 patients with LUAD, logistic regression analysis indicated that the expression level of *ADRB2* in LUAD was negatively correlated with gender (OR 0.603 for males vs. females, P = 0.004); smoking status (OR 0.457 for yes vs. no, P = 0.003); T classification (OR 0.581 for T2 vs. T1, P = 0.005; OR 0.308 for T3 vs. T1, P < 0.001; OR 0.293 for T4 vs. T1, P = 0.018); and pathologic stage (OR 0.527 for stage III vs. stage I, P = 0.011, Fig. 3).

### Impact of *ADRB2* on the prognosis of LUAD

**Figure 4:**
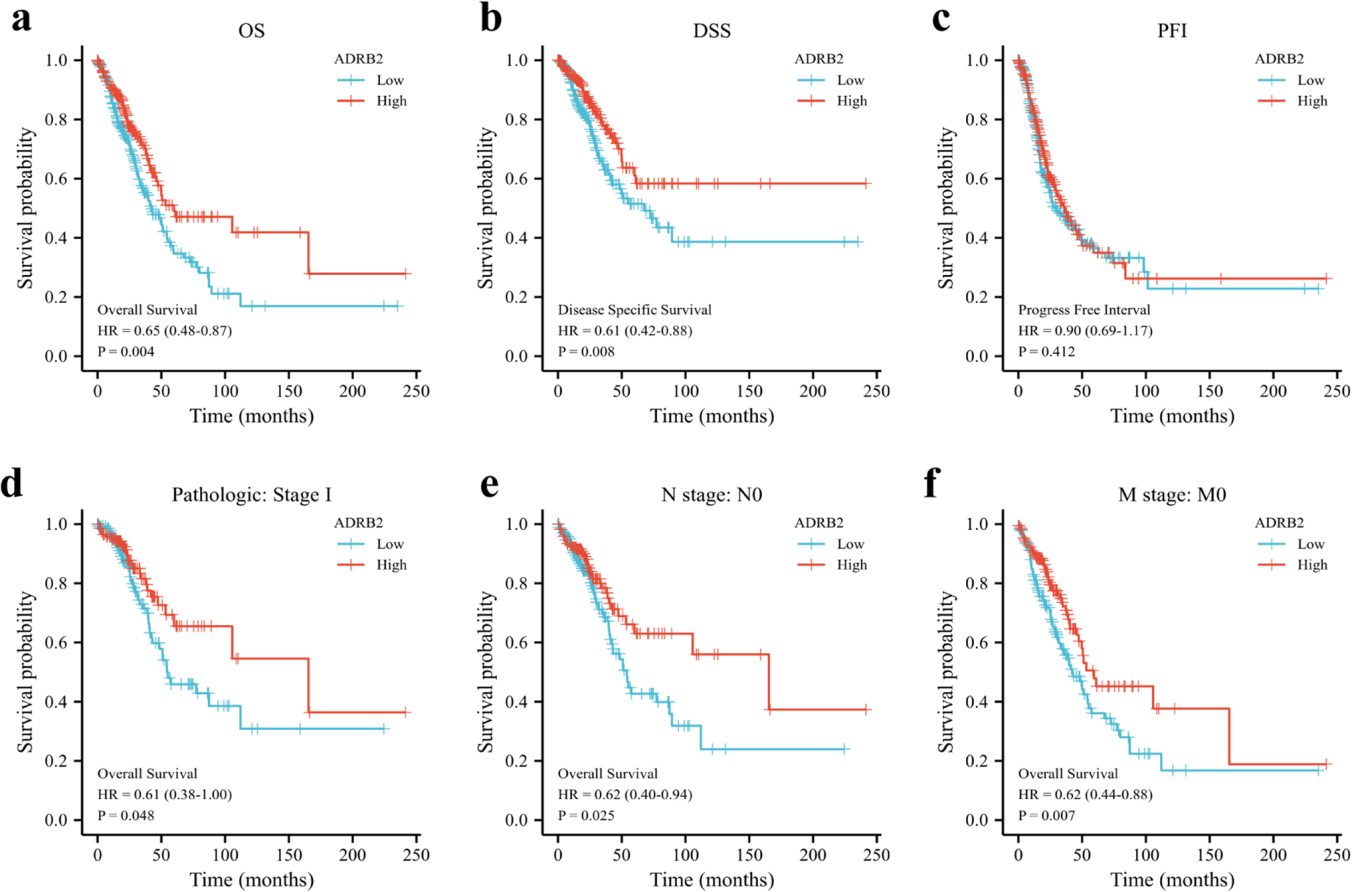
The prognostic value of *ADRB2* in the differernt subgroups. Survival analysis of *ADRB2* expression in LUAD patients: OS (**a**), DSS (**b**), and PFI (**c**). Low expression of *ADRB2* was associated with worse OS in different subgroups (**d**–**f**).

Survival curves were derived to assess the prognosis of high and low-*ADRB2* expression in LUAD patients. As displayed in Fig. 4, patients in the low *ADRB2* expression group presented with significantly poorer OS (HR 0.65(0.48–0.87), P = 0.004, Fig. 4a) and DSS (HR 0.61(0.42–0.88), P = 0.008, Fig. 4b) than those in the high *ADRB2* expression group. However, PFI did not differ between the two groups (HR 0.90(0.69–1.17), P = 0.412, Fig. 4c). Subgroup analysis suggested that the link between lower *ADRB2* expression and worse OS was statistically significant in most subgroups, especially in the stage I subgroup of pathologic stage (HR 0.61(0.38–1), P = 0.048), N0 subgroup of N stage (HR 0.62(0.40–0.94), P = 0.025), M0 subgroup of M stage (HR 0.62(0.44–0.88), P = 0.007).

### Effect of *ADRB2* expression on survival based on univariate and multivariate analyses

Univariate analysis revealed that pathological stage (HR 2.664; 95% CI 1.960–3.621; P < 0.001); T stage (HR 2.317; 95% CI 1.591–3.375; P < 0.001); N stage (HR 2.601; 95% CI 1.944–3.480; P < 0.001); M stage (HR 2.136; 95% CI 1.248–3.653; P = 0.006); and *ARDB2* expression (HR 0.612; 95% CI 0.456–0.821; P = 0.095) were meaningful indicators of survival, and multivariate Cox risk regression analysis showed that T stage (HR 1.817; 95% CI 1.119–2.951; P = 0.016) and N stages (HR 2.086; 95% CI 1.387–3.139; P < 0.001) were independent prognostic factors for OS (Table 2).

**Table 2:** Univariate and multivariate analyses of *ADRB2* and clinical pathological parameters associated with survival in patients with LUAD.

Parameter	Univariate analysis			Multivariate analysis		
	HR	95% CI	*P*	HR	95% CI	*P*
Age	1.223	0.916–1.635	0.172	1.256	0.881–1.792	0.208
Smoker	0.894	0.592–1.348	0.591	0.896	0.536–1.498	0.676
Gender	1.070	0.803–1.426	0.642	0.969	0.681–1.379	0.861
Pathological stage	2.664	1.960–3.621	< 0.001	1.341	0.805–2.233	0.260
T stage	2.317	1.591–3.375	< 0.001	1.817	1.119–2.951	0.016
N stage	2.601	1.944–3.480	< 0.001	2.086	1.387–3.139	< 0.001
M stage	2.136	1.248–3.653	0.006	1.263	0.654–2.450	0.490
*ADRB2*	0.612	0.456–0.821	0.001	0.730	0.504–1.056	0.095

### Evaluation of the diagnostic capacity of *ADRB2* in LUAD

**Figure 5:**
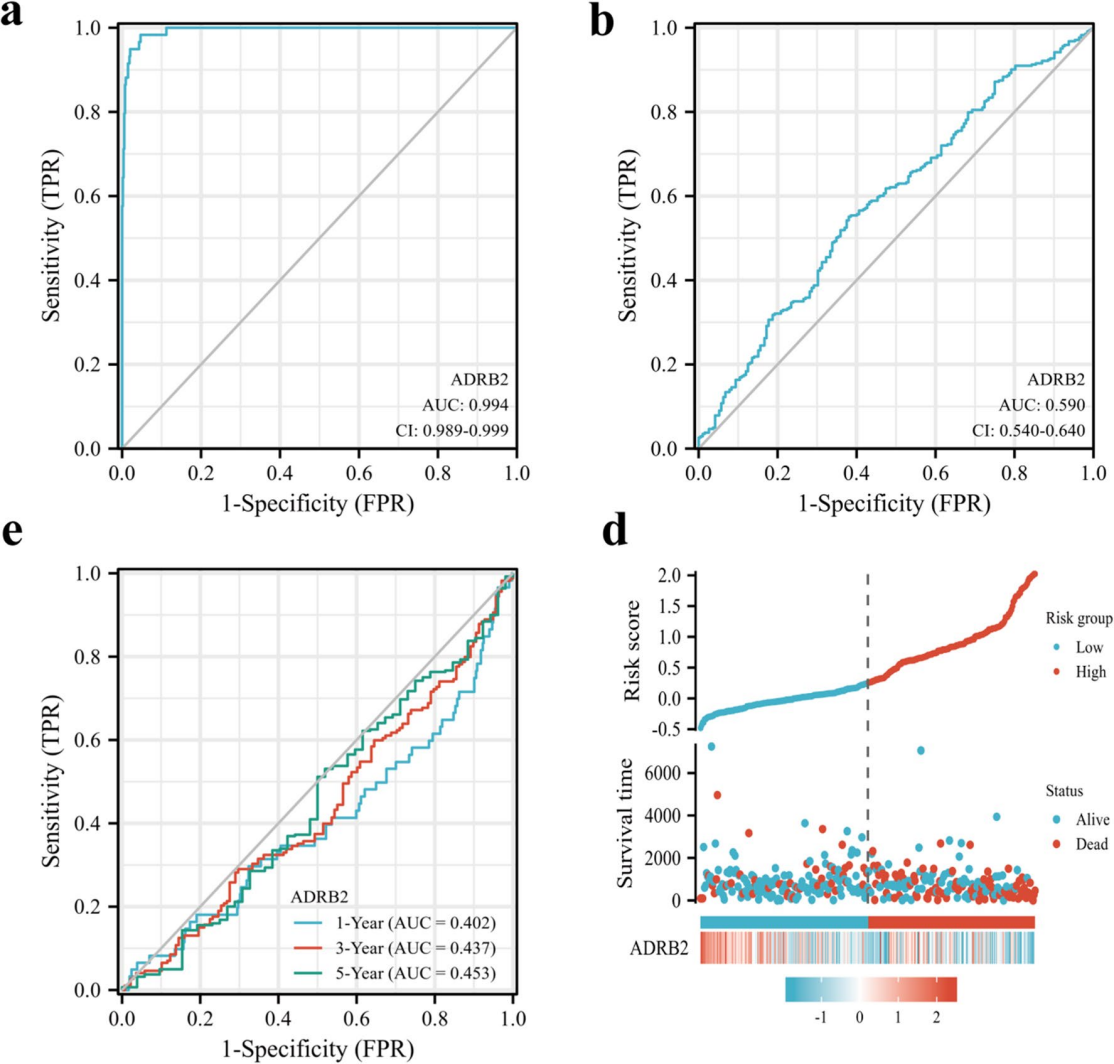
The prognostic value of *ADRB2* in LUAD. Diagnostic ROC curve of *ADRB2* (Normal vs. Tumor) (**a**). Prognosis ROC curve of *ADRB2* (Dead vs. Alive) (**b**). Time-dependent ROC curve of *ADRB2* (**c**). *ADRB2* expression distribution and survival status (**d**).

To explore the diagnostic value of *ADRB2* for LUAD, receiver operating characteristic (ROC) curve analysis was performed. The results of the ROC curves indicated that *ADRB2* was highly sensitive to the diagnosis of LUAD (AUC, 0.994; 95% CI 0.989–0.999, Fig. 5a). Additionally, the AUC was 0.598 for OS, which indicated that the prognostic model had good performance in predicting survival prognosis of patients with LUAD (AUC, 0.590; 95% CI 0.548–0.648, Fig. 5b). The time-dependent accuracy of *ADRB2* in predicting OS in 1, 3, and 5 years was also assessed through a time-dependent ROC analysis, AUC < 0.5 indicates that the expression of *ADRB2* is opposite to the occurrence trend of OS events in LUAD patients (Fig. 5c). Figure 5d shows that the survival rate of patients in the high-risk group is poor, and the risk of death is high. With the decrease of *ADRB2* expression, the risk score tend to increase gradually.

### Relationship of *ADRB2* expression level with immune infiltration in LUAD

**Figure 6:**
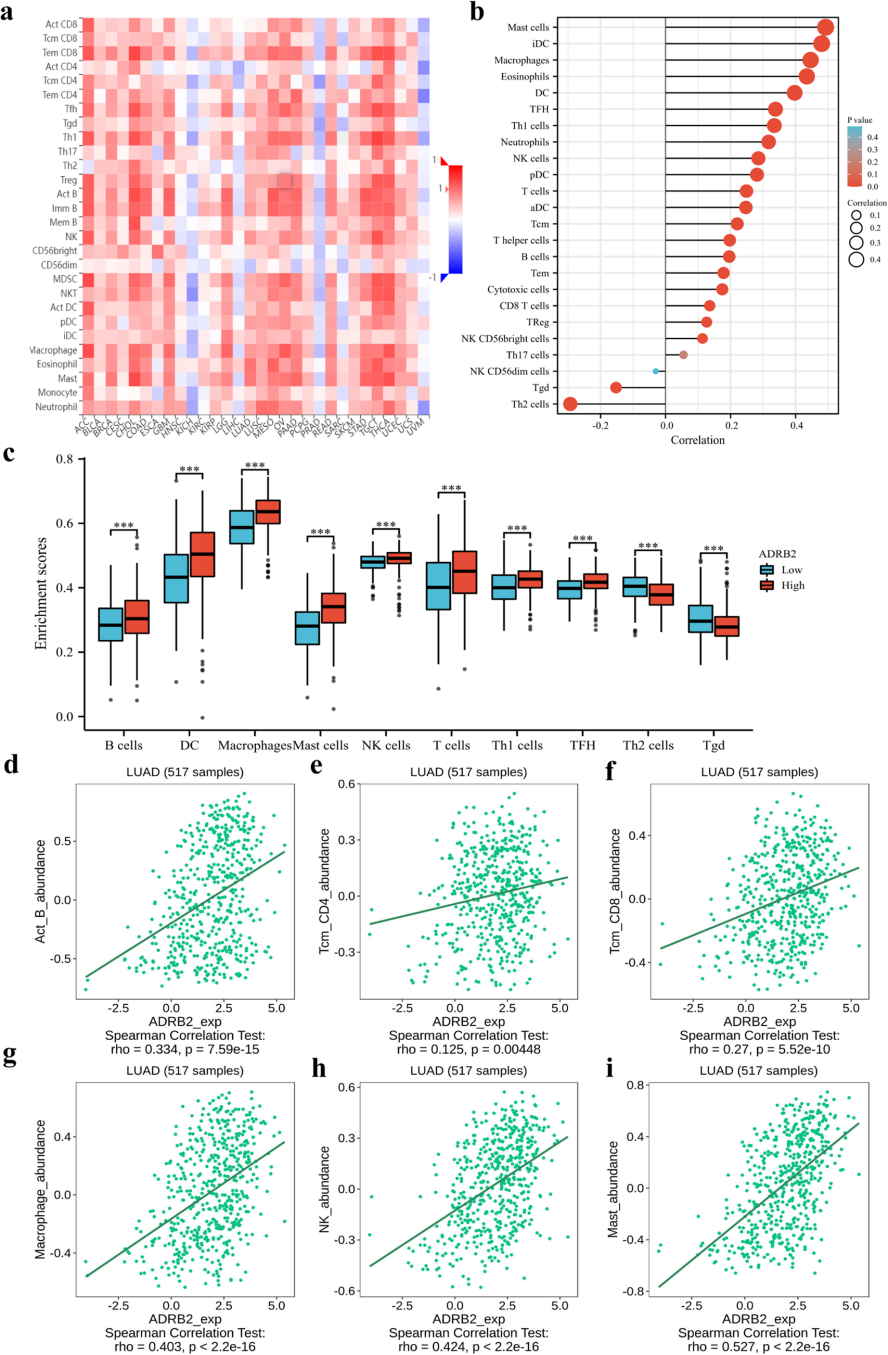
Relations between the expression of *ADRB2* and 28 types of TILs across human cancers (**a**). The relationship between immune cell infiltration and *ADRB2* expression (**b**). The infiltration levels of immune cell populations in lung adenocarcinoma (LUAD) patients with different *ADRB2* expression (**c**). *ADRB2* was correlated with abundance of B cells, CD4 T cells, CD8 T cells, macrophage, NK cells, and mast cells (**d**–**i**). *P < 0.05, **P < 0.01, ***P < 0.001.

Figure 6a shown the relationships between expression of *ADRB2* and 28 types of tumor-infiltrating lymphocytes (TILs) across human cancers in the TISIDB database. In TCGA database, pearson’s analysis demonstrated that the infiltration of 24 types of immune cells was markedly related to *ADRB2* expression, which had a significantly positive relationship with CD8 T cells (P = 0.049), type 17 Th cells (Th17) (P = 0.021), and regulatory T cells (TReg) (P = 0.025), and a strongly-positive association with activated DCs (aDCs), B cells, cytotoxic cells, dendritic cells (DCs), eosinophils, immature DCs (iDCs), macrophages, mast cells, neutrophils, natural killer (NK) cells, neutrophils, plasmacytoid DCs (pDCs), T cells, T helper cells, Tcm T central memory (Tcm), T effector memory (Tem), T follicular helper (TFH), and type 1 Th cells (Th1) (P < 0.001, Fig. 6b,c). However, T gamma delta (Tgd) and type 2 Th cells (Th2) (P < 0.001, Fig. 6b,c) showed a negative association with *ADRB2*. As can be seen in Fig. 6d–i, the expression of *ADRB2* was correlated with adundance of B cells (r = 0.334, P = 7.59e−15), CD4 T cells (r = 0.125, P = 0.00448), CD8 T cells (r = 0.27, P = 5.52e−10), macrophage (r = 0.403, P < 2.2e−16), NK cells (r = 0.424, P < 2.2e−16), mast cells (r = 0.527, P < 2.2e−16) in the TISIDB database.

### *ADRB2* associated gene set enrichment in LUAD

**Figure 7:**
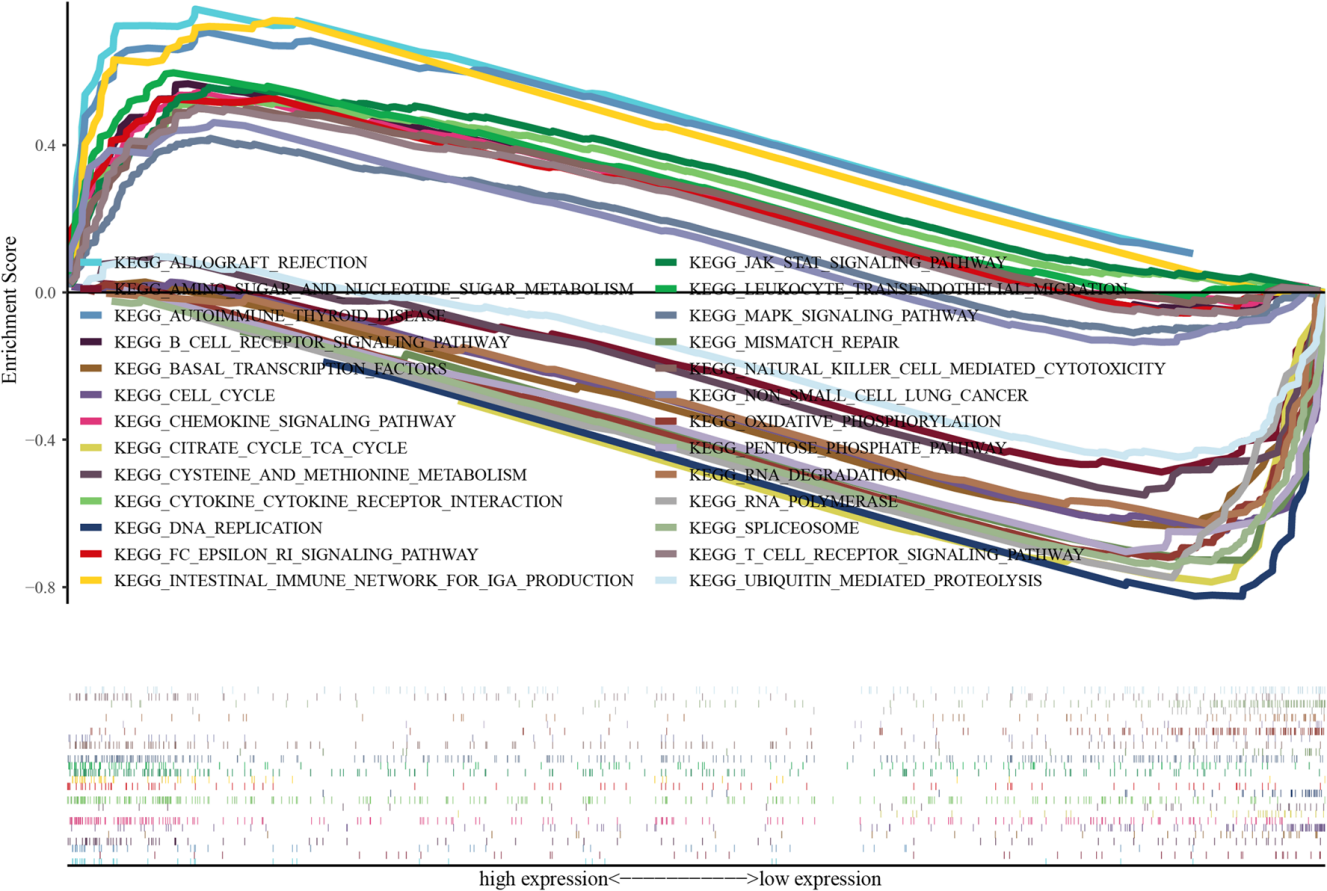
Enrichment plots from gene set enrichment analysis (GSEA).

To determine *ADRB2*-related signaling pathways, GSEA was performed between the high- and low-*ADRB2* groups. Significance was assessed using a normalized enrichment score (NES) ≥ 1.5, P ≤ 0.05, and false discovery rate (FDR) ≤ 0.25. KEGG pathway enrichment analysis indicated that 13 important signaling pathways were significantly enriched in the highly expressed *ADRB2* phenotypes, including the JAK STAT signaling pathways, leukocyte trans-endothelial migration, chemokine signaling pathway, autoimmune, thyroid disease, Fc epsilon ri signaling pathway, intestinal immune network for iga production, cytokine receptor interaction, B cell receptor signaling pathway, NK cell-mediated cytotoxicity, allograft rejection, Mapk signaling pathway, T cell receptor signaling pathway, and NSCLC. Meanwhile, there were 13 eligible signaling pathways enriched in the low-*ADRB2* expression, including spliceosome, RNA polymerase, RNA degradation, citrate cycle (or TCA cycle), cell cycle, pentose phosphate pathway, basal transcription factors, oxidative phosphorylation, DNA replication, mismatch repair, cysteine and methionine metabolism, ubiquitin-mediated-proteolysis, and amino sugar and nucleotide sugar metabolism (Table 3, Fig. 7). These results contribute to further exploration of *ADRB2* pathophysiological mechanisms.

**Table 3:** Gene sets enriched in the low and high *ADRB2* expression phenotypes.

Low expression				High expression			
Gene set name	NES	NOM p-value	FDR q-value	Gene set name	NES	NOM p-value	FDR q-value
KEGG_SPLICEOSOME	− 2.229	0	0	KEGG_JAK_STAT_SIGNALING_PATHWAY	2.170	0	0.004
KEGG_RNA_POLYMERASE	− 2.199	0	0	KEGG_LEUKOCYTE_TRANSENDOTHELIAL_MIGRATION	2.170	0	0.004
KEGG_RNA_DEGRADATION	− 2.183	0	0.001	KEGG_CHEMOKINE_SIGNALING_PATHWAY	2.037	0.003	0.010
KEGG_CITRATE_CYCLE_TCA_CYCLE	− 2.126	0	0.002	KEGG_AUTOIMMUNE_THYROID_DISEASE	2.036	0.002	0.008
KEGG_CELL_CYCLE	− 2.103	0.001	0.002	KEGG_FC_EPSILON_RI_SIGNALING_PATHWAY	2.019	0.001	0.009
KEGG_PENTOSE_PHOSPHATE_PATHWAY	− 2.075	0	0.003	KEGG_INTESTINAL_IMMUNE_NETWORK_FOR_IGA_PRODUCTION	2.009	0.004	0.009
KEGG_BASAL_TRANSCRIPTION_FACTORS	− 2.025	0	0.004	KEGG_CYTOKINE_CYTOKINE_RECEPTOR_INTER ACTION	1.996	0.007	0.010
KEGG_OXIDATIVE_PHOSPHORYLATION	− 1.998	0.003	0.005	KEGG_B_CELL_RECEPTOR_SIGNALING_PATHWAY	1.969	0.006	0.010
KEGG_DNA_REPLICATION	− 1.981	0	0.005	KEGG_NATURAL_KILLER_CELL_MEDIATED_CYTOTOXICITY	1.825	0.010	0.033
KEGG_MISMATCH_REPAIR	− 1.896	0.012	0.031	KEGG_ALLOGRAFT_REJECTION	1.825	0.012	0.031
KEGG_CYSTEINE_AND_METHIONINE_METABOLISM	− 1.798	0.001	0.025	KEGG_MAPK_SIGNALING_PATHWAY	1.821	0.001	0.031
KEGG_UBIQUITIN_MEDIATED_PROTEOLYSIS	− 1.743	0.014	0.037	KEGG_T_CELL_RECEPTOR_SIGNALING_PATHWAY	1.805	0.022	0.031
				KEGG_NON_SMALL_CELL_LUNG_CANCER	1.708	0.014	0.049

## Discussion

LUAD is a type of malignant lung tumor that originates from the bronchial mucosal glandular epithelium. LUAD is characterized by inconspicuous early symptoms, and LUAD is a lung tumor with a significant rate of malignant recurrence, metastasis, and unsatisfactory prognosis. Early diagnosis is difficult in most patients.

*ADRB2* is ubiquitously expressed in multiple tissues, including the smooth muscle of the human bronchi, cardiovascular system, central nervous system, and gastrointestinal tract. In recent years, increasing evidence shows that *ADRB2* has a vital place in the occurrence and development of diverse range of cancers. Zhang et al.^11^ found that the mRNA expressions of *ADRB2* were higher in gastric cancers compared with normal tissues. Moreover, patients with gastric cancer with positive *ADRB2* expression exhibited larger tumor size, late clinical stage, lower differentiation, and distant metastasis. In addition, high *ADRB2* expression can promote the angiogenic switch in prostate cancer and prevent or delay the dominant role of pro-angiogenic factors, leading to tumor progression^12^. β2-AR is encoded by *ADRB2* and can bind specifically to endogenous catecholamines (such as adrenaline and noradrenaline), and promotes the production and release of cyclic adenosine phosphate (cAMP). cAMP can further activate and phosphorylate protein kinase A and C to activate downstream signal transduction pathways and promote the proliferation, migration, and metastasis of lung cancer cells^13^. The positive *ADRB2* expression can occur in several cancers, including hepatocellular carcinoma, colorectal cancer, melanoma, and gastric cancer, and is often indicative of poor prognosis^5,11,14–16^. The regulation of β2-AR on tumorigenesis may be twofold, depending on the type of tumor and the stage of cancer progression. A study have revealed that activation of *ADRB2* results in inhibition tumor cell growth, as well as induction of apoptosis and tumor regression, where this activation results in the inactivation of the Raf-1/Mek-1/Erk1/2 pathway by a cAMP-dependent activation of protein kinase A^17^. In oral squamous cell carcinoma, patients with higher *ADRB2* had a significant longer DSS and OS^18^. Caparica et al.^19^ found that a high *ADRB2* expression may be a favorable prognostic factor in patients with HER2 + early breast cancer and evidence that a possible association with antiproliferative, antiangiogenic, and immunogenic effects of *ADRB2.* Yazawa et al.^20^ retrospectively analyzed 328 surgically-resected patients with NSCLC and found that positive *ADRB2* expression was found in 29% of LUAD tissues, which markedly increased compared with in non-adenocarcinoma tissues. A high level of *ADRB2* expression was associated with vascular invasion, tumor cell proliferation, and poor prognosis in patients with LUAD. Nevertheless, Wang et al.^10^ searched the gene expression synthesis (GEO) to obtain data showing that *ADRB2* is down-regulated in LUAD, and *ADRB2* mRNA levels declined with stage progression. *ADRB2* mRNA expression levels and its gene product, β2-AR, differ. The exact mechanism is still controversial. This study used high-throughput RNA sequencing data obtained from TCGA database to further ascertain the expression and the prognostic significance of *ADRB2* in LUAD and explore its correlation with immune cell infiltration.

The low expression of *ADRB2* in tumor tissues of LUAD patients is correlated with gender, smoking, OS events and DSS events, indicating that *ADRB2* is a potential biomarker for prognosis and diagnosis. Data mining from TCGA database showed that the expression of *ADRB2* was correlated with pathological stage, T stage and N stage, and the disease tended to progress with the decrease of *ADRB2* expression. Premised on this, logistic regression analysis showed that *ADRB2* is significantly correlated with gender, smoking status, T stage and pathological stage. The survival analysis revealed a favorable survival in high *ADRB2* expression group compared to those with low expression. Univariate analysis revealed that pathological stage, T stage, N stage, M stage, and *ADRB2* expression influenced OS. To further determine the diagnostic capacity of *ADRB2* in LUAD, ROC curves were used to confirm that *ADRB2* is sensitive to the diagnosis and prognosis of LUAD. Altogether, these findings illustrate that *ADRB2* is a potential prognostic biomarker for LUAD.

The tumor immune microenvironment (TME) is very important in cancer pathogenesis^21^. Immune cells are vital elements of the TME^22^. The correlation between *ADRB2* expression and the infiltration of 24 immunocytes was further explored to elucidate the mechanisms responsible for *ADRB2* to predict clinical prognosis. Further correlation analysis indicated that the infiltration of 19 immune cells was significantly associated with *ADRB2* expression. *ADRB2* expression was positively correlated with aDCs, B cells, CD8^+^ T cells, cytotoxic cells, DCs, eosinophils, iDCs, macrophages, mast cells, neutrophils, NK cells, pDCs, T cells, T helper cells, Tcm, Tem, TFH, Th1 cells, Th17 cells, and TReg. B cells are dominant in the progression of lung cancers^23,24^ and can be observed at the individual stages of carcinogenesis^25^. B cells can prolong the survival of cancer patients by inhibiting tumor progression and preventing metastasis. In addition, antibodies produced by B cells are essential mediators of tumor cell death^26^. Cytotoxic T lymphocytes (CTLs) are major players in antitumor immunity and can lead to apoptosis of cancer cells through a series of steps; therefore, high infiltration of CTLs is a favorable prognostic marker for many cancers. DCs are the most effective antigen-presenting cells to induce primary tumor immune response^27^, and in NSCLC patients, an increased DC count was significantly associated with an increase in DSS^28^. The role of macrophages in cancer progression is still controversial. Tumor-associated macrophages promote tumor progression by facilitating tumor stroma formation and angiogenesis^29^. In patients with NSCLC with prolonged survival, macrophage-infiltrating tumors are mainly of the M1 type^30^. Mast cells, which have cytotoxic effects on cancer cells, can enhance the immunity of patients with LUAD against cancer cells and improve their postoperative prognosis^31^. NK cells are cytotoxic and it is essential in the immune monitoring of cancers^32^. Carrega et al.^33^ found that in resected LUAD tissues, increasing numbers of infiltrating NK cells were associated with favorable patient survival outcomes. T cells are the most abundant monocytes infiltrating the NSCLCs^34^. T cells can secrete cytokines to inhibit tumor stroma formation and use cytotoxic molecules to kill epithelial nuclear stromal cells. Al-Shibli et al.^35^ reported that T cell infiltration is associated with better DSS, and T cells are an independent indicator of survival. T helper cells play an important role in cancer immunity by secreting cytokines^36^. Both Th1 and Th17 cells produce proinflammatory factors, and their extensive infiltration can significantly improve clinical outcomes in a variety of cancers^37,38^. Large infiltration of cytotoxic T cells in tumor tissues is associated with longer survival^35^. Studies have shown that TFH has an antitumor response, and IL-21 secreted by TFH induces the activation, proliferation, and differentiation of B cells^39,40^. *ADRB2* expression may up-regulate the levels of infiltrating immune cells to limit the development of LUAD. In contrast, *ADRB2* expression was negatively correlated with Th2 cells and Tgd. Th2 cells have many pro-neoplastic activities and take part in cancer progression by cytokine release. Th2 cells are dominant in lymphocytes from malignant pleural effusion in patients with lung cancer^41,42^. Current studies have revealed that Tgd has a pro-tumor effect, which can inhibit innate and adaptive immunity by inducing immunosenescence^43–45^.

To summarize, low *ADRB2* expression is associated with poor prognosis of LUAD. These results show that *ADRB2* expression level affects the immunity activity in the TME, and *ADRB2* might be a valuable biomarker for the immune status in LUAD patients.

To further explore the mechanism of *ADRB2* in LUAD, the signaling pathways involved in *ADRB2* was screened. In the *ADRB2* high-expression group, *ADRB2* associated genes were significantly enriched in immune signaling pathways (such as B cell receptor signaling pathway, T cell receptor signaling pathway, and NSCLC, NK-cell-mediated cytotoxicity, chemokine signaling pathway, and Jak STAT signaling pathway), in KEGG analysis. Those are significant in the tumorigenesis, development, and invasion of malignancies^46^. On the other hand, in the *ADRB2* low-expression group, *ADRB2* correlated genes were enriched in metabolism-related pathways, including RNA polymerase, citrate cycle, pentose phosphate pathway, oxidative phosphorylation, cysteine and methionine metabolism, and amino sugar and nucleotide sugar metabolism, implying that *ADRB2* up-regulated the signaling pathways associated with immune response and induced antitumor efficiency. Therefore, *ADRB2* expression was down-regulated as LUAD progressed, and the TME switched from an immune-active state to a metabolic state. The *ADRB2* expression can be considered a biomarker to predict immune response.

At present, most studies on the relationship between *ADRB2* and the occurrence and progression of LUAD are based on its gene expression product, β2-AR, and its signaling pathway. This study revealed *ADRB2* as a key gene in the immune microenvironment of LUAD by performing a bioinformatics analysis, to provide evidence for *ADRB2* as a potential prognostic marker for LUAD. However, some limitations arise in the research. Firstly, the present research was limited by the small number of cases, and a large cohort is needed to validate the results of this research. Secondly, this research primarily focused on the expression of *ADRB2* mRNA from TCGA, and without involving β2-AR levels in LUAD tissues. Thus, this study still needs large-sample, multi-center, multi-ethnic clinical trials and basic experimental studies to prove the prognostic value of *ADRB2* in LUAD. Low expression of *ADRB2* is associated with poor prognosis in LUAD patients, which may be related to immunocompromised.

## Conclusion

In summary, *ADRB2* expression was significantly down-regulated in patients with LUAD. *ADRB2* is involved in LUAD progression partly by regulating the immune microenvironment, which may potentially serve as a significant prognostic biomarker as well as a potential drug target.

## Materials and methods

### Data acquisition

On or before November 13, 2021, the mRNA profile (HTSeq-Counts and HTSeq-FPKM) was extracted from TCGA (https://cancergenome.nih.gov/), including 535 LUAD samples and 59 normal samples. Relevant clinical information was derived from TCGA. RNAseq data in FPKM (Fregments Per Kilobase Per Million) format were converted to TPM (transcripts Per Million reads) format and log2 transformed. *ADRB2* expression data from datasets GSE40791 was downloaded from the GEO database (https://www.ncbi.nlm.nih.gov/gds/). The relevant data TCGA and GEO provided is open-access, no additional approval from the Ethics Committee were required. All methods were performed in accordance with the relevant guidelines and regulations.

### *ADRB2* expression and survival analyses

The original expression data downloaded from TCGA were processed using the Perl programming language (version: strawberry-perl-5.32.1.1-64bit.mis, https://strawberryperl.com/). GEOquery package (version: 2.54.1, https://cran.r-project.org/web/packages/GEOquery/index.html) is used to download GEO database expression data. The differential *ADRB2* expression were analyzed by Mann–Whitney U test, Dunn’s test, Kruskal–Wallis test or Spearman correlation analysis when appropriate, and the results were visualized using the ggplot2 R package (version: 3.3.2, https://cran.r-project.org/web/packages/ggplot2/index.html). Binary logistics regression model was used to evaluate the relationship between *ADRB2* and clinical characteristics. Survival data were extracted and analyzed using the Perl programming language, and patients without complete survival state and time were removed. Subsequently, we matched the complete survival data with *ADRB2* expression data and obtained 499 patients’ data. In survival analysis, the *ADRB2* mRNA expression level was split into two groups by the median expression value, and OS, DSS, and progression-free interval (PFI) were evaluated with cox regression and log-rank test. A Kaplan–Meier survival curve was constructed by the survival (version:3.2-10 https://cran.r-project.org/web/packages/survivalAnalysis/index.html) and survmine (version:0.4.9,https://cran.r-project.org/web/packages/survminer/index.html) package of R software. pROC R package (version: 1.17.0.1, https://cran.r-project.org/web/packages/pROC/index.html) and timeROC R package (version:0.4,https://cran.r-project.org/web/packages/timeROC/index.html) were used for statistical analysis, and ggplot2 R package was used for visualization when drawing ROC curves and risk score map.

### Univariate and multivariate Cox regression analyses

Both univariate and multivariate analyses of clinical pathological parameters were performed adopting Cox proportional hazards analysis. In addition, we quantitatively evaluated the independent predictive value of clinicopathological parameters and *ADRB2* expression for survival and explored the prognostic effect of *ADRB2* on survival after adjusting for other confounding factors. Meanwhile, when matching it with *ADRB2* expression data, incomplete clinical information was excluded.

### Evaluation of immune infiltration

Figure 6a is drawn online in TISIDB (http://cis.hku.hk/TISIDB/index.php). GSEA method from the R package “GSVA (version:1.34.0, http://bioconductor.org/packages/release/bioc/html/GSVA.html), clusterProfiler (version:3.18.0, http://bioconductor.org/packages/release/bioc/html/clusterProfiler.html), and rtracklayer (http://bioconductor.org/packages/release/bioc/html/rtracklayer.html)” was used to present infiltration enrichment of 24 common immune cells in each sample, including mast cells, DCs, iDCs, macrophages, eosinophils, TFH, Th1, neutrophils, pDCs, T cells, NK cells, B cells, aDCs, Tem, T helper cells, cytotoxic cells, Tcm, CD8 T cells, TReg, NK CD56 bright cells, Th17, NK CD56dim cells, Tgd, and Th2. After that, Pearson’s analysis was used to investigate the relationship between *ADRB2* expression level and 24 immune cell infiltration in LUAD. Pearson correlation test and the independent-samples T test were used to compare the levels of immune cell infiltration between different *ADRB2* expression groups. Spearman’ test was used to evaluate the relationship between *ADRB2* expression and the abundance of TILs.

### Gene set enrichment analysis (GSEA)

All LUAD patients in TCGA dataset were allocated into high and low group based on the expression of *ADRB2*. GSEA was used as a signaling pathway analysis tool to explore the signaling pathways related to *ADRB2* in LUAD. GSEA between high and low *ADRB2* expression was performed using GSEA 3.0 (http://www.gsea-msigdb.org/gsea/index.jsp). Phenotypes were determined based on *ADRB2* expression levels. The gene set “c2 all.v6.0 symbols.gmt” was used for the enrichment analysis. KEGG analysis was performed to explore the significant pathways associated with *ADRB2* expression.

### Statistical analysis

All statistical analyses were performed using IBM SPSS Statistics for Windows, version 23.0 (IBM Corp, Armonk, NY, USA) and R (version 4.0.2, https://www.r-project.org/) and the level of statistical significance was defined as a P < 0.05.

## Supplementary Information

Supplementary Table S1.

## Data Availability

The datasets generated and/or analysed during the current study are available in TCGA dataset (https://cancergenome.nih.gov/) and GEO database (https://www.ncbi.nlm.nih.gov/gds/).
