# Supplementary material for: ADRB2 expression predicts the clinical outcomes and is associated with immune cells infiltration in lung adenocarcinoma
Source: Sci Rep. 2022 Sep 26;12:15994. doi: 10.1038/s41598-022-19991-y (PMC9512930; doi:10.1038/s41598-022-19991-y)
Supplement: Supplementary file 1 — Supplementary Table S1. [file 41598_2022_19991_MOESM1_ESM.pdf]

ADRB2 expression predicts the clinical outcomes and is associated with immune cells infiltration in lung adenocarcinoma

Lingyun Ji[1#] Fei Xu[2#] Jingtao Zhang[3] Ting Song[2] Weida Chen[2] Xi Yin[2]
Qingqing Wang[4] Xiubao Chen[2] Xin Li[2] Minghao Guo[2] Zetao Chen[2*]

Supplement Table 1: Correlation analysis between *ADRB2* expression and clinicopathological features

Characteristic	Spearman correlation	P
Pathological stage	-0.165	**0.002**
T stage	-0.237	**0**
N stage	-0.128	**0.019**
M stage	-0.049	0.371
